# Web-based screening and brief intervention for poly-drug use among teenagers: study protocol of a multicentre two-arm randomized controlled trial

**DOI:** 10.1186/1471-2458-12-826

**Published:** 2012-09-26

**Authors:** Nicolas Arnaud, Sonja Bröning, Magdalena Drechsel, Rainer Thomasius, Christiane Baldus

**Affiliations:** 1 Centre for Psychosocial Medicine; German Centre for Addiction Research in Childhood and Adolescence, University Medical Centre Hamburg-Eppendorf, Martinistraβe 52, Hamburg, D-20246, Germany

## Abstract

**Background:**

Mid to late adolescence is characterised by a vulnerability to problematic substance use since the consumption of alcohol and illicit drugs is frequently initiated and increased in this life period. While the detrimental long- and short-term effects of problematic consumption patterns in adolescence pose a major public health concern, current prevention programs targeting alcohol- and other substance-using adolescents are scarce. The study described in this protocol will test the effectiveness of a web-based brief intervention aimed at reducing problematic alcohol use and promoting abstinence from illegal drugs in adolescents with risky substance use aged 16 to 18 years old in four EU-countries.

**Methods/design:**

To determine the effectiveness of our web-BI, we apply a two-arm randomized controlled trial (RCT) study design, with baseline assessment at study entry and a three month follow-up assessment. Adolescents aged 16 to 18 years from Belgium, the Czech Republic, Germany, and Sweden will be randomly assigned to either the fully electronically delivered brief intervention group (*N* = 400) or an assessment only control group (*N* = 400) depending on their screening for risky substance use (using the CRAFFT). Recruitment, informed consent, randomization, intervention and follow-up will be implemented online. Primary outcomes are reductions in frequency and quantity of use of alcohol and drugs other than alcohol over a 30 day period, as well as consumption per typical occasion. Secondary outcomes concern changes in substance use related cognitions including the constructs of the Theory of Planned Behaviour, implementation intentions, and stages of change. Moreover the study addresses a number of moderator variables, including age of first use, general psychopathology and quality of parent–child relationship.

**Discussion:**

The trial is expected to contribute to the growing literature on theory- and web-based brief interventions for adolescents. We will explore the potential of using web-based technologies as means of delivering preventive interventions. In doing so we are among the first to target the relevant group of young poly-drug users in Europe.

**Trial registration:**

Current Controlled Trials ISRCTN95538913

## Background

Mid to late adolescence constitutes a phase of major developmental changes and age-specific demands associated with the biographic transition into a more adult role [1-3]. While the hallmarks of adolescence are exploration of identity and future prospects [4,5], this phase in the lifespan is also characterized by a certain vulnerability to substance consumption [6] since the consumption of alcohol and illicit drugs is frequently initiated and increased in this life period [7,8].

The European School Survey Project on Alcohol and Other Drugs (ESPAD) periodically reports prevalence rates among students in 35 European countries. Overall, this report demonstrates a considerably high proportion of substance using adolescents particularly concerning early and heavy drinking. In 2007, an average of 90% of the 15–16 year old students in Europe had consumed alcohol at some point in their life. Most of them started to drink alcohol at the age of 13 years [9]. An average of 50% of European students experienced alcohol-intoxication at least once in their lifetime and 39% did so during the last month. Moreover, one of three European students had smoked cigarettes during the past 30 days and one of five had used cannabis at least once in his or her life, which was also true for 7% of the students regarding illicit drugs other than cannabis. Apparently substance use is rarely restricted to the consumption of a single substance as illicit drug consumption is most typically accompanied by alcohol [9]. Such poly-drug patterns appear prevalent among youth in modern Europe as emphasized in the 2009 Annual report by the European Monitoring Centre for Drugs and Drug Addiction [10].

Consumption of various substances (and their combinations) in adolescence is associated with severe adverse effects on psychological and physiological development [11,12]. Untimely (i.e., early) and hazardous use of alcohol, cannabis and other substances in early years puts youth at risk for developing chronic problematic consumption patterns that can significantly influence their developmental trajectories during the transition from child- to adulthood [13,14]. Detrimental long term effects of establishing chronic problematic consumption patterns and substance use disorders (SUDs) and the associated health problems are apparent [15]. Also, there are serious short-term consequences related to substance consumption such as accidents, vandalism, risky sexual behaviour [7], delinquency, truancy, problems of academic adjustment [12] and problems with the police or legal authorities [16]. Beyond the immense personal risks involved, problematic substance use also imposes significant economic burdens on the European Union in terms of health care costs, which translate into an estimated amount of 20 billion Euro per year caused by alcohol use in Germany alone [17]. In light of these negative consequences substance use can be qualified as a large risk potential and mayor public health concern in Western societies [18,19].

The high prevalence of substance use in Europe is a problem that has been recognized in the EU Drug Strategy 2005–2012 [20]. To date, however, in Germany as in the European Union, current prevention programs targeting alcohol- and other drug-using adolescents (i.e., early indicated prevention) that meet scientific evaluation criteria are scarce. The study described in this protocol aims at filling this gap and will test the effectiveness of a web-based brief intervention aimed at reducing alcohol use and promoting abstinence from illegal drugs among adolescents with risky substance use aged 16 to 18 years.

### Screening and brief interventions

To date, Brief Motivational Interventions are the most empirically supported individual level interventions for reducing alcohol use [21]. This approach is based on principles of Motivational interviewing (MI) such as empathic and reflective listening in a non-judgemental and non-confrontative style [22]. It also draws on the literature on social influence [23], with the goal of increasing discrepancy and motivation to change consumption behaviour. Previous studies have demonstrated the effectiveness of web-based brief interventions that rely entirely on a fully automatised and electronically delivered brief intervention (i.e., requiring direct real-time contact with an interventionist) particularly for problematic alcohol [24,25] and – albeit sporadically - cannabis use [26]. Existing studies have included different contexts, such as prevention of young adults’ hazardous alcohol consumption in the workplace [27] but typically focus on the problem of college drinking [24,25,28]. Although the therapeutical potential of internet-based interventions for substance abuse problems has been widely recognised [29-31], little research has addressed how effectively this approach generalises to an adolescent population. This notwithstanding, first results are encouraging. Newton, Andrews, Teeson, and Vogl [32] using a cluster randomised controlled trial found that 13-years old students participating in an internet based prevention program reduced their average weekly drinking and frequency of cannabis use six months after the intervention compared to the control group. Concerning underage and adolescent drinking, Spijkerman and colleagues [33] have recently demonstrated the effectiveness of a multi-component web-based brief intervention (i.e., providing personalized feedback about drinking pattern, drinking motives, health risk status, and advice on reduced drinking) targeting binge drinkers aged 15 to 20 years in a RCT. Overall, they found that a single-session exposure of approximately 15 minutes duration was associated with decreases in weekly drinking. Interestingly, effects were more pronounced in males showing less weekly alcohol use and higher levels of moderate drinking (consumption of less than 14 alcoholic drinks in the past week for males, less than 7 for females) over a 1 to 3 month period. These initial results match with established findings in the literature that traditional face-to-face brief intervention approaches in health care and treatment settings [34] for adolescents are associated with substantial decreases in substance use [35-38].

Although apparently more studies are required since health-related behaviour change using the internet in general is still in the early stages of development [39], recent experimental and systematic reviews suggest that brief interventions delivered entirely via computer programs [40,41] or the internet [42-44] match the efficacy of person-delivered interventions [45]. At the same time they offer a number of advantages over conventional methods. Specifically, they are easily implemented and disseminated, and they provide the opportunity to reach a large audience by improved access to interventions in a very cost effective manner [46,47]. In addition, they maintain participants’ privacy and anonymity [28], and provide flexible access at any time and location, the latter of which is especially relevant for areas lacking service possibilities. Furthermore, web-based approaches appear particularly suitable to a younger target group for which internet use is very common [48]. Nowadays, adolescents prefer the internet as means of finding information about drugs [49], and might be more willing to disclose alcohol- and drug-related behaviour in a web-delivered format because it is less stigmatising [50] and requires less problem awareness in order to be accepted [51]. This latter aspect is especially relevant for younger people, among whom denial of mental health problems is common [52].

Given these benefits, it is not surprising that computerised intervention approaches regarding substance use figure prominently in current literature [42,50]. However as Copeland and Martin [29] note, overall this literature is largely descriptive and lacks controlled trials, in particular with respect to adolescent populations. Moreover to date the literature has not adequately addressed possible mechanisms of change inherent to MI-approaches using theoretically derived constructs [53,54] and lacks a more fine-graded description of specific subgroups that benefit from such an approach (i.e., moderators).

#### Aim

Considering the promising results and the apparent benefits of electronically delivered screening and brief intervention for people with hazardous substance consumption, together with the need to investigate new ways of approaching the important target group of poly-drug using adolescents in Europe, this study primarily aims at creating a fully automatised web-based brief intervention for substance-using adolescents in four European countries and examine its effectiveness across a range of outcome measures. Secondary research questions concern potential predictors, mediators and moderators of positive outcomes.

## Method/design

**Figures 1:**
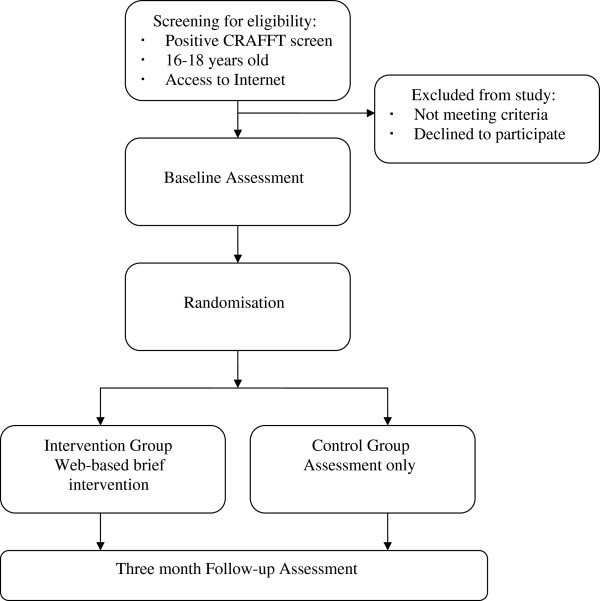
Study design.

The relevant target group for this study are adolescents aged 16 to 18 years from Belgium, the Czech Republic, Germany, and Sweden. To determine the effectiveness of the web-BI, we apply a two-arm RCT study design, with baseline assessment at study entry and a three month follow-up assessment. Figure 1 displays the trial design.

### Recruitment

In contrast to other studies that test web-based interventions offline before putting them on the internet [28,55] we will launch Czech, German, Flamish, and Swedish versions of our website and motivate potential participants to visit by running online (using social networks and banner advertisements) and offline marketing campaigns. Similar to others [28] we will rely on a convenience sample from the general population. To attract adolescents, motivate them to participate and keep their attention, our intervention will be constructed to be personalised, playful and interactive, featuring an attractive graphical design and a presentation mode that factors in age-specific and gender-sensitive aspects. The aim is to generate a highly dynamic intervention that contrasts to other more informational and static web-portals. Additionally, to enhance follow-up rates, a sweepstake for participants successfully completing follow-up assessment is provided in each country.

### Study flow

The landing page will provide five language flags (languages of the four participating countries plus an English Version) for choosing a different language than the language pre-defined by browser options. Those first visiting the page will be asked for registration which requires a user name, e-mail address and a pass word. After registration respondents will be presented with a developmentally appropriate screening tool for adolescents to examine if they fulfil the inclusion criterion of a substance problem. For screening we rely on an adapted version of the CRAFFT^a ^[56] for use of alcohol and drugs. This tool uses six items and has proven criterion validity and appropriateness for identifying substance-related problems among adolescents [57] compared to other screening tools [58]. A CRAFFT score higher than 1 (i.e., at least two questions are answered with yes) demonstrates sensitivity to identify substance problems [58] and is deemed sufficient to be eligible for participating in the study. Besides a positive screen for substance-related problems, participants must be between 16 and 18 years of age. Participants matching the inclusion criteria will then receive study information including confidentiality advice,^b^ the indication of voluntariness of participation, and of human subject protections [28]. They are then asked to electronically give informed consent. Approval for the design and data collection was already obtained from the responsible authorities in each participating country, which are the Ethical Committee of the Chamber of Physicians Hamburg (Germany), Prague Psychiatric Centre (Czech Republic), University Hospital of Antwerp and the University of Antwerp (Belgium), and the Regional Ethics Board in Stockholm (Sweden).

### Assessment

Those eligible and willing to participate in the study will receive comprehensive baseline assessment questionnaires containing socio-demographic characteristics, such as age, gender, educational/vocational information, as well as their weight which will be the base for an individualised and personalised feedback in the later intervention (weight is necessary to calculate Blood-alcohol-concentration). Items concerning current substance consumption (primary outcomes) and cognitive items concerning substance use (secondary outcomes) will be assessed at baseline and follow-up. A number of potential moderator variables are included in the baseline assessment to allow for subgroup analyses. All measures are based on established scales or self-constructed according to the literature (see *Instruments* section). All assessment material will be pilot-tested for appropriateness and duration as completing the survey should not take long in order to avoid attrition.^c^

### Randomisation

Randomisation will take place after completion of the screening procedure, informed consent statement and baseline questionnaires. Random allocation to either the intervention or the control group (assessment only) will be generated automatically by an online computer program and cannot be influenced by researchers. No stratification procedure is included since the expected number of participants is sufficient to make a balanced distribution among the two parallel groups likely [59]. Randomisation checks will be conducted on baseline behavioural and psychological measures using multivariate analyses of variance (MANOVA) with consumption and consumption-related cognitions as dependent variables and intervention condition (brief intervention vs. control) as the independent variables [60]. Participants will be informed of their allocation with those in the intervention condition receiving a log-in code if they wish to exit and re-enter at a later point in time.

### Intervention

#### Theoretical background

Our intervention relies on models of Social Influence [23,61] and Motivational Interviewing [22], which have proven effectiveness in prior relevant research [34,62]. As described earlier, Motivational Interviewing is a client-centered, non-judgmental, non-confronting approach to decrease drinking and drinking-related consequences [22], which is well-suited for adolescents [63,64]). The goal of an MI approach is to enhance motivation to change by exploring and resolving ambivalence about current substance-related behaviour. Important elements are the presentation of discrepant personal information by providing individualised feedback about substance consumption behaviour. The latter will be relative to normative comparisons in our intervention, balancing pros and cons of the target behaviour and supporting self-efficacy. Provision of individualised feedback regarding risk-status and normative feedback relative to a relevant comparison group figure prominent in the literature on web-based brief interventions [40] since “tailored” feedback is presumably perceived as more relevant than more general information [28]. Normative feedback involves information about how a specific reference group actually consumes in order to correct adolescents’ “inflated perception” [21] and of the amount the reference group typically consumes. It has been proven effective for reduction of alcohol consumption in young adults in recent meta-analyses [65,66]. For young people, a normative feedback approach may be particularly appealing assuming high levels of curiosity in how their substance consumption compares to their peers [27]. Likewise, an important element of our approach is a focus on substance-related social norms and training on how to avoid social high-risk situations and resist peer pressure (i.e., raising refusal self-efficacy) [31,67]. In line with the social influence hypothesis, which states that one of the most prominent risk factors for substance use in adolescence is the influence of substance using peers [68], targeting peer pressure in brief web based interventions has been integrated in recent web-based interventions for reducing alcohol use among adolescents [28].

#### Description of the intervention

The overarching goal of the intervention is to encourage reduced alcohol consumption and abstinence from any illicit drugs.^d^ The single-session intervention is fully automatised and electronically delivered yet interactive. It works with presenting tailored feedback to the participants’ responses in the earlier assessment (i.e., consumption levels) and provides choice options on how to react to this feedback. This interactivity simulates a face-to face “dialogue”, which aims for an empathic style, avoids argumentation, rolls with resistance and aims at creating a dissonance between actual and desired behaviour and raising self-efficacy. To achieve these goals, our intervention basically consists of three components. First, participants will receive personalised feedback on their substance consumption patterns including the associated risks (related to health and other consequences) and comparisons to a normative reference group. Second, participants engage in interactive MI-based exercises that have been proven effective in prompting readiness to change by encouraging the participant to consider the costs and benefits of their current substance use and actual change. Finally, the intervention will include practical advice concerning alternative behaviour in tempting situations, with a focus on peer resistance skills to raise self-efficacy beliefs and implementation intentions. All components will be described in more detail below. The whole intervention can be completed in approximately 10 to 15 minutes.

##### Feedback

Individualised feedback includes evaluation of consumption patterns (e.g., frequency and quantity of use and binge-drinking) concerning various substances according to the answers given in the assessment part. Risk status for negative consequences associated with drinking, use of illicit drugs, and polydrug use (i.e., combined use of various substances) will be based on the participant’s Alcohol (Drug) Use Disorder Identification Tests (AUDIT-C and DUDIT, see Instruments section below). Furthermore, feedback includes an estimation of the blood-alcohol-concentration (BAC) and information on the associated risks (health and other risks, such as traffic crash, unintended sex etc.) concerning the participants’ heaviest drinking episode during the past 30 days. This value will be based on a measure of *Peak Drinking Quantity*[69] (see *Instruments*) and can be estimated using the Widmark-formula which is sensitive for weight and gender [70]. Finally, participants will receive individualized and graphed feedback regarding the following information: a bar chart comparing the number of glasses of standard alcohol units per week that participants think their peers actually consume (descriptive norms), as well as the participant’s individual levels of drinking, both in relation to comparative data (actual drinking levels) from a reference group. Comparative data (AUDIT-C scores) will be taken from alcohol prevalence estimates found in a nationally representative samples of 18 year old males in Sweden.^e^ Comparative feedback will be available for drinking but not for drugs other than alcohol.

##### Interactive MI-based exercises

Based on the literature on MI [22,71], generally the exercises provided in WISEteens assume that 1) participants may hold certain levels of ambivalence about their current substance use (i.e., they can see the advantages and disadvantages of drinking and/or taking drugs), and 2) if they are willing to make a change they may not know how to make a change or may not be confident that they are able to make a change. Based on this assumption, we use importance and confidence rulers with a short summary and feedback to encourage participants to think about their possible personal reasons for change and explore their personal strengths and their ability to change. Furthermore, we implement a decisional balance [72] to pick up and graphically illustrate potential levels of ambivalence by offering the participants a list of possible pros and cons regarding the decision to change their current substance use [73]. Participants are instructed to choose those statements that apply to them and are presented with the resulting balance sheet of their personal comparative potential gains and losses.

##### Practical Advice (menu of options)

In addition to targeting confidence for change in the exercises described above we will focus on raising self-efficacy for avoiding drinking in social situations and providing practical advice to resist temptations. Similar to the approach of Voogt and her colleagues [28] we will ask participants to select three among 12 provided drinking situations that are chosen as most tempting and rank them (the situations are adapted from the adolescent version of the Drinking Refusal Self-efficacy Questionnaire (DRSEQ-RA) [74]. According to the selection several tips will be offered for each of the selected drinking situations to provide participants with a tool kit necessary for engaging in and maintaining their behavioural goal (that is reduced drinking and / or abstain from illegal drugs). Again, practical advice refers to resistance skills for drinking, but participants are encouraged to apply the advice given (e.g., peer resistance) also for resisting drugs other than alcohol and to avoid risky situations in a similar way.

### The control group: assessment only

The control group will receive no intervention besides the assessment. However, participants in the control group are provided with contact information on suitable counselling service providers to get in touch with in case of severe substance use-related problems. All participants are invited to visit again and participate in follow-up assessment after the evaluation period of the intervention of 3 months.

### Instruments

#### Primary outcomes

The primary outcomes of the study refer to reductions of substance use. As prevalence rates of alcohol are particularly high among youth we distinguish measures for alcohol, which will be the main focus, from those in the broader category of drugs other than alcohol. Specifically we use the Alcohol Use Disorder Identification Test Consumption subscale (AUDIT-C) [75]) for frequency of alcohol consumption, frequency of having five drinks (four for girls) at one occasion (binge drinking), with response options ranging from 1 (*never*) to 5 (*four or more times a week*). Rather than using the third original AUDIT-C item, which measures number of standard drinks consumed per typical drinking occasion (1 = *one or two* to 5 = *ten or more*), and in order to ensure standardised responses across the four participating countries, we use a graphical overview of various types of drinks with the indication to select the number of each drink per typical drinking occasion. This way we can better account for national differences in typical standard drinks while keeping the content of the item. Standard drinks are defined as 10 g ethanol and items are assessed over the past 30 days.

For drugs other than alcohol we analogously use the first three items of the Drug Use Disorder Identification Test (DUDIT) [76] assessing frequency of consumption of drugs other than alcohol, frequency of different types of drugs other than alcohol used at the same occasion (1 = *never* to 5 = *four or more times a week*), and quantity of consumption of drugs other than alcohol on a typical consumption day (1 = *zero* to 5 = *seven or more*).

While changes in AUDIT-C and DUDIT scores are the primary outcome measures, the study includes other behavioural measures as well. From the AUDIT-C indication of frequency, quantity and selection of drinks during the last 30 days we will be able to calculate and possibly observe changes in *Weekly Alcohol Consumption* (in gram), which will be matched against the national recommended limits of each country (e.g., for Germany according to published guidelines this limit is 24gr/week for males and 12gr/week for females [77]. In addition we will measure *Peak Drinking Quantity* by asking participants to indicate the amount of drinks consumed on the occasion on which they drank the most during the last month [69], *Frequency of Drinking to Intoxication* (“*During the past 30 days, how many times have you gotten drunk, or very high from alcohol?*”, with response option ranging from *0* to *9 or more*) [27], and *Typical Weekend Drinking*, following Doumas and Hannah [27] by using a modified version of the Daily Drinking Questionnaire [78] using the following item: “*Given that it is a typical week, please write the number of drinks you probably would have each day”*; (*Monday*_______, *Tuesday*______, etc.). Weekend drinking will be the combined reports for Friday and Saturday.

#### Secondary measures

In addition to the behavioural outcome variables described above we assess a number of potential and theoretically plausible moderator and mediator variables, as well as several cognitive-level outcomes. Specifically, we assess the following moderators: *Age of first use*, *General Psychopathology*, using a short version of the Symptom Checklist (SCL-K 9) [79] and *Child Disclosure*[80], as an indicator of parent–child relationship quality. Positive parent–child relationship is a protective factor for adolescent substance use [81] and has been consistently shown to be related to lower likelihood of adolescent substance use [82] and variables related to adolescent substance use, such as self-control, competence, and peer affiliations [83]. Regarding potential mediators and cognitive outcomes we rely on socio-cognitive constructs based on major psychological models predicting health behaviour change, such as Ajzen’s Theory of Planned Behaviour (TPB) [84], and Prochaska & Di Clemente’s Transtheoretical Model (TTM) [85,86]. Both theories are widely used in clinical practice and health psychology and generalise across many different problem behaviours [87,88]. Specifically, we address *attitudes*, *subjective norms*, *perceived behavioural control*, and *behavioural intentions* as the proximal cognitive antecedents of goal-directed behaviour. Additionally we include a measure of *Implementation Intentions*[89] to address the action planning phase in goal attainment. Implementation intentions are defined as plans that specify the conditions under which target behaviours will be performed and help to ensure that the decision is acted upon [90]. Moreover, we include an algorithm for allocating participants into different *stages of change* concerning their substance use [91-93] hypothesising that progression (such as from precontemplation to contemplation) will be more pronounced in the intervention group compared to the control group. All items are adapted from previous related research.

### Sample size

Sample size is based on a power calculation for detecting a small effect size of *d* = 0.2 according to Cohen [94] . Obviously, our hypotheses are directional with better outcomes expected for the intervention group. Following Cohen’s convention [94] the trial is powered to detect a small effect size of *d* = 0.2 or larger given errors of 1−*β* = .80 and *α* = .05 in a one-sided test [95]. Expecting a worst-case scenario of 50% loss to follow-up rate, we aim to recruit n = 200 participants per country, totalling to 800 respondents (n = 400 per condition).

### Statistical analyses

To test the efficacy of WISEteens we address the primary research question whether participation in the intervention leads to a decrease in drinking and / or drug consumption other than alcohol at follow-up after three months compared to those adolescents in the assessment-only control group. The main analysis will follow separate analyses for alcohol and drugs. For alcohol consumption we will run multivariate analyses of covariance (MANCOVA) with intervention condition as the independent variable (intervention vs. control group) on the primary dependent variables frequency and quantity (i.e., number of drinks per typical drinking occasion) of alcohol consumption, frequency of binge drinking (AUDIT-C), reductions in weekly drinking and peak drinking. Baseline alcohol units of these variables and change scores (baseline-follow-up) of motivational (i.e., implementation intentions) and the socio-cognitive constructs (i.e., intentions, attitudes, subjective norms, PBC) based on Ajzen’s Theory of Planned Behaviour (TPB) [84] will be included as covariates. For drug use other than alcohol we will analogously run separate analyses of variance (MANCOVAs) on the primary dependent variables frequency and quantity of drug use (DUDIT-C). In addition, we will test whether socio-demographic variables (age, sex, social class), country of residence (Sweden, Germany, Belgium, Czech Republic), general psychopathology, parent–child relationship, age of first use, and stage of motivation to change moderate the outcomes. Moreover we will explore possible mediated effects of our intervention on the various behavioural measures relying on multiple mediation analysis using the syntax of Preacher and Hayes [96] for SPSS, and test direct and indirect effects in a path model using AMOS 18 [97]. Path Analysis is a multivariate technique specifying relationships between observed variables akin to running several regression equations with the advantage that it allows simultaneous testing of several propositions regarding how constructs are theoretically linked and the directionality of significant relationships within one single model [98].

## Discussion

The present study protocol describes a two-arm RCT examining the effectiveness of a web-based brief intervention for adolescents aged 16 to 18 in four European countries. The fully electronically delivered intervention is built upon the principles of MI [22] and is explicitly designed to promote reductions in frequency and quantity of problem drinking and abstinence of drugs other than alcohol. The scope of our research questions includes theory-based analyses of the mechanisms involved in the expected behaviour change (i.e., mediators) as well as à priori specification of subgroups for differential analyses (moderators).

Internet-based brief interventions hold a number of significant advantages over traditional clinician-delivered approaches and have proven effective in addressing alcohol and drug use in the general population. Initial results concerning adolescent target groups are promising yet scarce, even more so in the field of poly-drug use. The present study will contribute to the literature on web-based interventions for this particular target group.

The present study has a number of strengths. First and foremost, our intervention is well grounded in theory incorporating elements of Motivational Interviewing and social influence which has been shown to be effective in reducing problematic substance consumption in prior research. Also, our research is guided by major psychological models of health related behaviour change, such as Ajzen’s Theory of Planned behaviour [84] and the Transtheoretical model [85]. In our study we will account for the articulated need to better understand the underlying factors of MI approaches and investigate the ways in which health behaviour may be changed to enhance effectiveness of (MI-based) interventions in promoting health [54,99]. Furthermore we are informed by the current state of the art in web-based intervention studies. For example we follow a tailored feedback approach which is likely to yield higher levels of acceptance than more static approaches especially for adolescents.

Among the limited amount of studies using adolescent samples to our knowledge we are the first to design and test a brief intervention for poly-drug using adolescents in a fully-electronic format. Another strong aspect of the present trial is its multi-centre approach with collected data from four European countries, allowing for international comparisons. Finally, we consider it strength to rely solely on internet recruitment in our target population. This approach matches real conditions and directly tests the external validity of our website should its effectiveness be proven. Note that this procedure deviates from other studies which typically test offline before going online [28,55]. While failure to retain a sufficient proportion of participants for follow-up assessment remains a concern for the evaluation we consider it vital to develop a web-site that will be accepted by our target group.

## Conclusions

In this study protocol, we described a research project for testing an intervention aimed at reducing drinking and promoting abstinence from illicit drugs among adolescents in four European countries. Evaluation of the intervention will provide insights into the effectiveness of web-based screening and brief intervention for poly-drug use among adolescents aged 16 to 18 years in Europe.

## Endnotes

^a^CRAFFT represents an acronym of the first letters of the key words addressed in the test’s six items (Car, Relax, Alone, Forget, Friends, Trouble).

^b^As a part of this participants will be encouraged to create a new e-mail address, one that does not contain their name.

^c^Note that the amount of assessment tools necessary for this evaluation study exceeds the amount of items necessary for running the later internet version of the intervention, and will be removed after the evaluation phase of the project.

^d^Note that we focus particularly on drinking. Nevertheless, participants will be encouraged throughout the intervention to take the exercises as templates for use of other substances.

^e^Because of large heterogeneity in the format of the national prevalence data we decided to use the Swedish representative national AUDIT-C scores as the basis for normative comparisons.

## Competing interests

The authors declare that they have no competing interests.

## Authors’ contributions

CB, NA designed the study and its methodology, CB coordinates the study and NA drafted the manuscript of this paper. MD participated in designing the study and carrying through its organisational processes and cooperations. SB conceived of the study and obtained funding. RT was involved in all parts of the article and the project as general supervisor of the research group. All authors read and approved the final manuscript.

## Pre-publication history

The pre-publication history for this paper can be accessed here:

http://www.biomedcentral.com/1471-2458/12/826/prepub
